# Polyimides as Promising Cathodes for Metal–Organic Batteries: A Comparison between Divalent (Ca^2+^, Mg^2+^) and Monovalent (Li^+^, Na^+^) Cations

**DOI:** 10.1021/acsaem.3c00969

**Published:** 2023-06-27

**Authors:** Damien Monti, Nagaraj Patil, Ashley P. Black, Dionysios Raptis, Andreas Mavrandonakis, George E. Froudakis, Ibraheem Yousef, Nicolas Goujon, David Mecerreyes, Rebeca Marcilla, Alexandre Ponrouch

**Affiliations:** † Institut de Ciència de Materials de Barcelona (ICMAB-CSIC), Campus UAB, 08193 Bellaterra, Catalonia, Spain; ‡ Electrochemical Processes Unit, IMDEA Energy, Avda. Ramón de La Sagra 3, 28935 Móstoles, Spain; § MIRAS Beamline, ALBA Synchrotron Light Source, Carrer de la Llum 2-26, 08290 Cerdanyola del Vallès, Spain; ∥ POLYMAT University of the Basque Country UPV/EHUAvenida Tolosa 72, 20018 Donostia-San Sebastián, Spain; ⊥ ALISTORE−European Research Institute, CNRS FR 3104, Hub de l’Energie, 15 Rue Baudelocque, 80039 Amiens, France; # Department of Chemistry, University of Crete, Voutes Campus, GR-71003 Heraklion, Crete, Greece; ∇ Centre for Cooperative Research on Alternative Energies (CIC energiGUNE), Basque Research and Technology Alliance (BRTA), Alava Technology Park, Albert Einstein 48, 01510 Vitoria-Gasteiz, Spain

**Keywords:** battery, lithium, post Li, sodium, calcium, magnesium, organic cathode

## Abstract

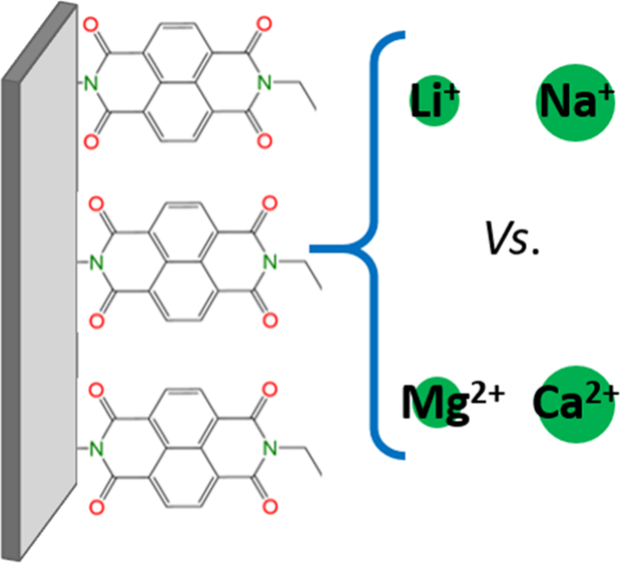

Ca- and Mg-based batteries represent a more sustainable alternative to Li-ion batteries. However, multivalent cation technologies suffer from poor cation mass transport. In addition, the development of positive electrodes enabling reversible charge storage currently represents one of the major challenges. Organic positive electrodes, in addition to being the most sustainable and potentially low-cost candidates, compared with their inorganic counterparts, currently present the best electrochemical performances in Ca and Mg cells. Unfortunately, organic positive electrodes suffer from relatively low capacity retention upon cycling, the origin of which is not yet fully understood. Here, 1,4,5,8-naphthalenetetracarboxylic dianhydride-derived polyimide was tested in Li, Na, Mg, and Ca cells for the sake of comparison in terms of redox potential, gravimetric capacities, capacity retention, and rate capability. The redox mechanisms were also investigated by means of operando IR experiments, and a parameter affecting most figures of merit has been identified: the presence of contact ion-pairs in the electrolyte.

## Introduction

Modern societies need a large panel of energy sources including intermittent renewables where batteries are anticipated to be the key players driving global energy transition ambitions. Independent of the application (e.g., e-mobility or stationary energy-storage system), current and future battery technologies have to fulfill energy/power density agendas, cost efficiency, safety checks, and, most importantly nowadays, sustainability requirement.^1^ Even if Li-ion batteries using conventional inorganic intercalation compounds and carbonate electrolytes are still dominating the current market, debates on the forecasted scarcity of lithium^2^ and the harmful environmental impact of conventional battery cathode materials (Co, Mn, Ni, and V)^3,4^ prompted the development of alternative materials and technologies. One of the plausible alternative is to develop Na ion^5,6^ or even divalent batteries, that is, magnesium and calcium batteries.^7−9^ The raw materials involved in these post-Li batteries are commonly more abundant, cheaper, and arguably less toxic.^10^ Moreover, the possible use of metal anodes could lead to breakthrough in terms of energy density.^11^ However, the lack of operational electrolyte and cathode materials for multivalent batteries has considerably slowed down the progress in the field, although encouraging advances have been lately reported.^7,12^ In this context, organic electrodes are promising sustainable candidates for next-generation multivalent batteries.^13−17^

Although already investigated in the early 70s, organic cathodes have been sidelined for decades because of the limited performance of the first developed polymers^18,19^ and by the tremendous success of inorganic electrodes from the 90s.^20^ This past decade, however, the performances of intercalation electrode were pushed closely to their theoretical limits and environmental concern has dramatically increased, which have somehow triggered the renewed interest in organic electrodes.^13^ Although they used to be disregarded for their low energy density, short cycle life, and high self-discharge, the recent development of new organic materials (e.g., radical polymers, redox-active polymers, etc.) with tunable properties represents a breakthrough showing that organic electrodes could provide long-term cycle life (low solubility), high capacity, and high voltage. The most studied organic compounds are listed as conductive polymers,^21,22^ organosulfur compounds,^23^ oxynitride radical compounds,^24,25^ imine compounds,^26^ and carbonyl conjugated compounds.^27,28^ Polyimides (PIs), belonging to the carbonyl family, are one of the most promising redox-active materials for batteries.^29−32^ Typically, PIs have multiple electroactive functional groups involving either aromatic or aliphatic structures that are conjugated to carbonyl groups. It is generally accepted that upon reduction, the oxygen anion of the carbonyl coordinates with the cation charge carriers through enolation mechanism,^33^ and each unit can reversibly transfer up to 2 moles of electrons per mole of repeating unit, rendering theoretical capacities ranging between 150 and 200 mAh g^–1^.^32^ In addition, as they stem from natural biomass extracts, they are expected to be inexpensive and easy to produce, which are interesting assets for the development of sustainable cathodes.^13^ Lately, reports on PI cathodes have provided promising figure of merits in monovalent (Li^+^, Na^+^, and K^+^-ion) organic batteries.^30,32,34,35^ Although a wide range of analytical tools have been used to demonstrate the redox mechanism (incl. electrochemical performance),^36−38^ spectroscopic analyses are sparse. Moreover, to the best of our knowledge, PI cathodes have not been extensively studied using multivalent cations, particularly divalent Ca ion in organic electrolytes.

In this study, we evaluate the electrochemical performance of 1,4,5,8-naphthalenetetracarboxylic dianhydride (NTCDA)-derived polyimide (PNTCDA) as the positive electrode for Li^+^, Na^+^, Mg^2+^, and Ca^2+^ ion batteries in organic electrolytes. A detailed comparison is made in terms of redox potential, gravimetric capacities, capacity retention, and rate capability. Moreover, operando IR-assisted experiments on Li^+^ and Ca^2+^ ion cells allow us to observe the reversible enolation/carbonylation processes of the carbonyl bonds in the imide functionalities in real time.

## Experimental Details

Electrolytes were prepared by mixing LiTFSI (99%), NaTFSI (99.9%), Ca(TFSI)_2_ (99.5%), or Mg(TFSI)_2_ (99.5%) purchased from Solvionic in an ethylene carbonate (EC, anhydrous 99.0%, Aldrich) and propylene carbonate (PC, anhydrous 99.7%, Aldrich) mix of EC:PC (1:1 wt %). Salt concentrations were set to 1.0 and 0.5 M, respectively, for monovalent and divalent salts unless specified. The water content in the electrolytes was measured by Karl-Fisher titration and found to be lower than 20 ppm in all cases. Electrolyte preparation and cell assembly were always carried out inside an argon-filled glovebox with <1 ppm H_2_O and O_2_.

The cathode material NTCDA-derived PNTCDA was synthesized by a polycondensation reaction between NTCDA and ethylenediamine as described previously.^35^

The electrochemical stability of the electrolytes was evaluated in three-electrode Swagelok cells using 316 L stainless-steel plungers, oversized activated carbon (AC) cloth (Kynol, ACC-509220) counter electrodes (CEs), and sulfonated Ag wires as reference electrodes.^39^ In order to prepare the reference electrodes, silver wires were first scratched using a sand paper and then sonicated in acetone and rinsed with distilled water. Then, they were immersed attached to a stainless-steel plunger in a solution of 5% NaSO_4_ for 24 h yielding a black coating of Ag_2_S covering the entire silver wire. Finally, the whole reference was dried overnight at 60 °C. The working electrode (WE) was a self-standing blend of PNTCDA active material, single-walled carbon nanotubes (SWCNTs), and reduced graphene oxide (RGO) in the proportion of 60:20:20 wt % (PNTCDA:SWCNT:RGO) with an average PNTCDA mass loading of 2.5 mg cm^–2^ (see the Supporting Information for the detailed electrode preparation). The cycling protocols always started with an equilibration step of the WE for 3 h at an open-circuit potential. In order to monitor the rate capabilities and specific capacities, cells were cycled using a Bio-Logic VMP3 potentiostat in the galvanostatic mode with potential limitation (GCPL) between 1C and C/20, 1C being normalized to the insertion of 2 moles of electron per hour (the upper and lower cutoff potentials were 0 V and −1.0 or −1.2 V vs Ag/Ag_2_S, respectively). Cyclic voltammetry (CV) was carried out at 5 mV s^–1^ between 0.7 and −1.2 V vs Ag/Ag_2_S for 30 cycles in the same Swagelok three-electrode configuration as described in the GCPL protocol.

Scanning electron microscopy (SEM) studies were performed using a Quanta 200 ESEM FEG FEI microscope equipped with an energy dispersive X-ray detector with an energy resolution of 132 eV. Samples were transferred from an argon-filled glovebox with minimum air exposure.

Operando Fourier transform infrared (FTIR) spectroscopy was conducted at the MIRAS beamline of ALBA synchrotron light source (Cerdanyola del Vallès, Spain) using a 3000 Hyperion microscope coupled to a Vertex 70 spectrometer (Bruker, Germany). The spectra were collected with a mercury-cadmium-telluride detector using the internal source of radiation. The microscope optics used a 36× Schwarzschild objective (NA = 0.52) with an aperture size of 50 × 50 cm^2^. The measurements were performed in a ECC-Opto-Std (EL-CELL) equipped with a 0.3 mm thickness CaF_2_ window. Electrochemical tests were performed on the self-standing PNTCDA [(60:20:20), (PNTCDA:SWCNT:RGO)] electrodes employing 1.0 M LiTFSI or 0.5 M Ca(TFSI)_2_ in EC:PC as electrolytes. Li metal and AC cloth (Kynol, ACC-509220) were used as CEs in Li and Ca cells, respectively. Cells were cycled in the GCPL mode at C/10 rates using a Bio-Logic SP-200 potentiostat.

## Results and Discussion

### Vibrational Spectroscopy

**Figure 1:**
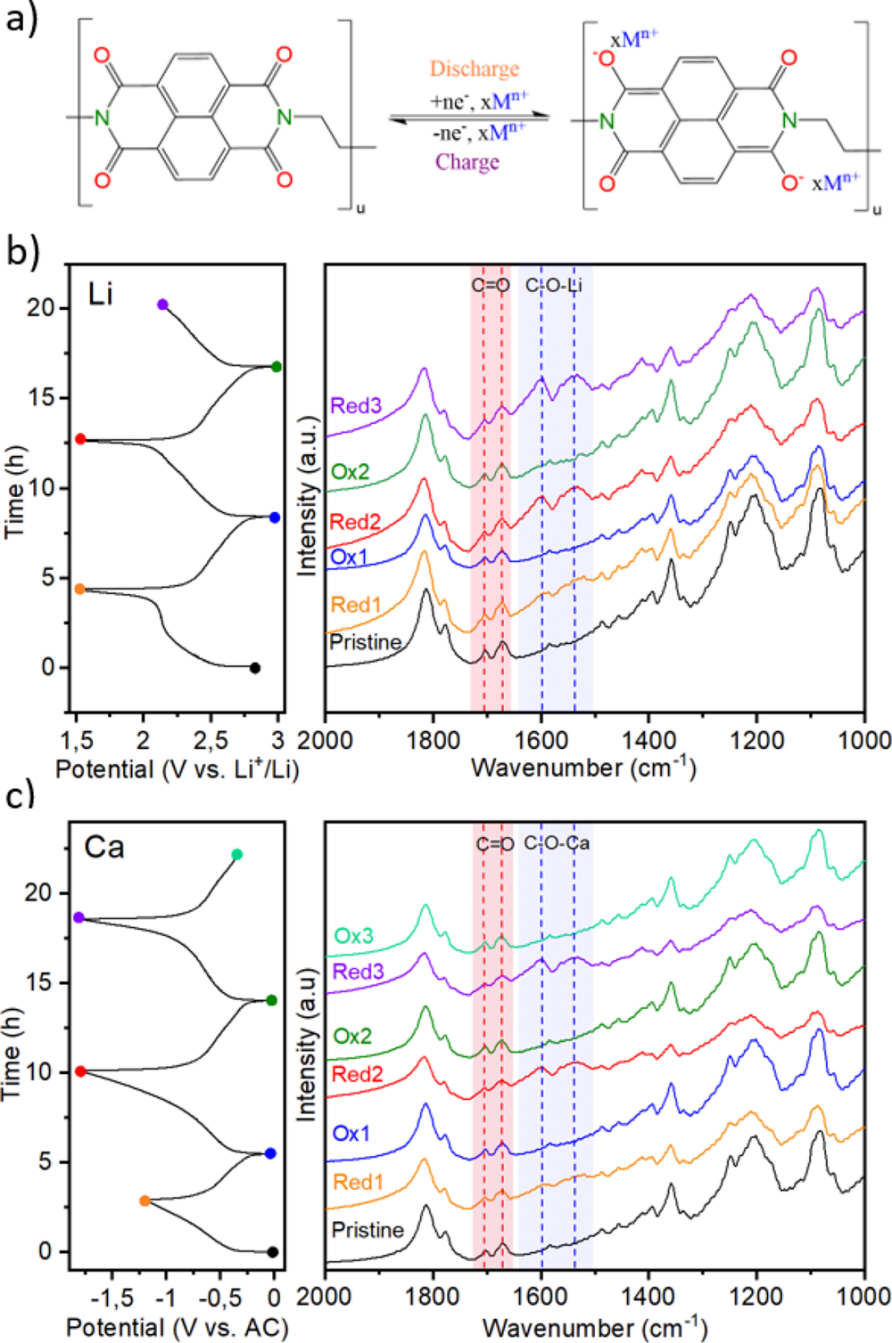
(a) Proposed charge/discharge mechanism of PNTCDA; M^*n*+^ = Li^+^ or Ca^2+^. The GCPL profiles of PNTCDA at C/10 in 1 M LiTFSI in EC:PC (b) and in 0.5 M Ca(TFSI)_2_ in EC:PC (c) with their corresponding IR spectra in the 2000–1000 cm^–1^ (*operando* measurements).

Operando FTIR spectroscopy was performed in order to observe the evolution of all infrared active bands and assess the reversible mechanism for the conversion of redox-active carbonyl groups in the imide functionality of PNTCDA via enolation/carbonylation reactions. The redox reaction of the PNTCDA is paired with the coordination and uncoordination of metal ions during discharging/charging processes (Figure 1a). The proposed mechanism was strongly inspired by redox reactions already reported for both aromatic polyimides and polyquinones.^30^ The commercial EL-CELL setup allowed us to collect the IR spectra while running GCPL measurements at C/10 between 3 and 1.5 V vs Li^+^/Li (Figure 1b) and between 0 and −1.75 V vs AC for the Ca cell (Figure 1c) using 1 M LiTFSI (Figure 1a) and 0.5 M Ca(TFSI)_2_ in EC:PC, respectively. Detailed operando FTIR spectra are given in the Supporting Information (Figure S1). We used a color code between the GCPL profile and IR spectra to individually distinguish the end of each oxidation and reduction and plotted them successively in Figure 1b,c. First, all spectra exhibit bands of the solvent species, that is, EC and PC, at about 1850–1750 cm^–1^ assignable to the stretching modes of C=O.^40^ On the other hand, characteristic bands of PNTCDA are located in the 1720–1300 cm^–1^ region.^32^ C=O asymmetric and symmetric stretching modes of the imide electrode are found, respectively, at 1703 and 1673 cm^–1^ (highlighted in red in Figure 1b,c). The naphthalene ring distortion is found at 1579 cm^–1^, while the C–N stretching mode is found at 1355 cm^–1^. Upon cycling, similar behavior is observed for both Li and Ca cells. Briefly, while no significant decrease in the intensity of the bands associated with the C=O asymmetric and symmetric stretching modes is observed, two new unassigned strong bands appear upon reduction located at 1600 and 1520 cm^–1^ (highlighted in blue in Figure 1b,c). They are weakly visible at the end of the first reduction and much more prominent for the two following reduction steps, suggesting some type of activation taking place during the first cycle. The cyclic appearance of these bands indicates that the process is reversible and we assume that it is associated with the formation of C–O^–^–M^+^ (M^+^ = Li^+^, Ca^2+^) bands similar to reports on anthraquinone redox reactions.^16^ No significant difference in the position of these two new broad bands can be seen between Li and Ca cells (see Figure S2).

As expected, both C–O^–^–M^+^ and C=O bands are present at the end of reductions which indicates that a proportion of C=O remains unreacted. Indeed, even if the electrode is totally reduced incorporating two electrons per naphthalene-imide ring, there will be still two additional C=O groups that are not redox active and thus their respective IR band will appear in the spectra. Unfortunately, the low intensity of the bands associated with the naphthalene ring distortion and the C–N stretching does not allow further conclusion regarding other functional groups of the PNTCDA. The operando FTIR spectra of Ca cells (Figure S1) present a cyclical increase and decrease of the spectra background that is correlated with the state of charge and reaching its maximum at the end of each reduction. This can be due to a change in the focus associated with the swelling and contraction of the electrode upon cycling, pointing at a more important swelling of the organic positive electrode in Ca than in Li cells. We tentatively ascribe this difference to the fact that the Ca^2+^ solvation shell being significantly larger than the one of Li^+^, with more solvent molecules,^41^ could result in more important swelling of the polymer during cation uptake.

### Electrochemistry

**Figure 2:**
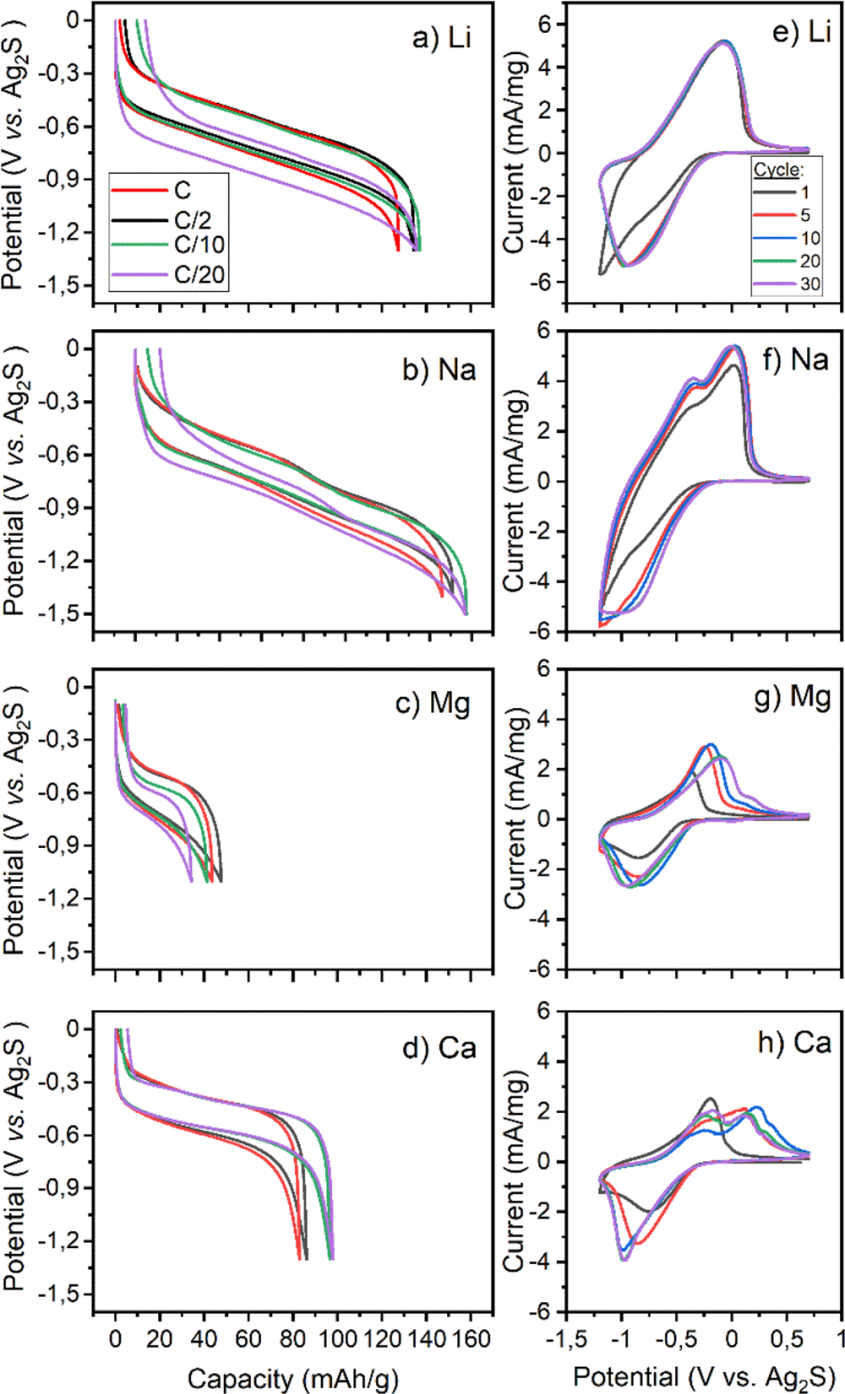
GCPL potential (vs Ag/Ag_2_S) vs capacity curves of PNTCDA in 1 M (a) LiTFSI and (b) NaTFSI and 0.5 M (c) Mg(TFSI)_2_ and (d) Ca(TFSI)_2_ in EC:PC for the last cycles at 1C, C/2, C/10, and C/20 rates and (e, h) CVs (cycles 1, 5, 10, 20, and 30 at 5 mV/s) obtained using the same electrolytes.

**Figure 3:**
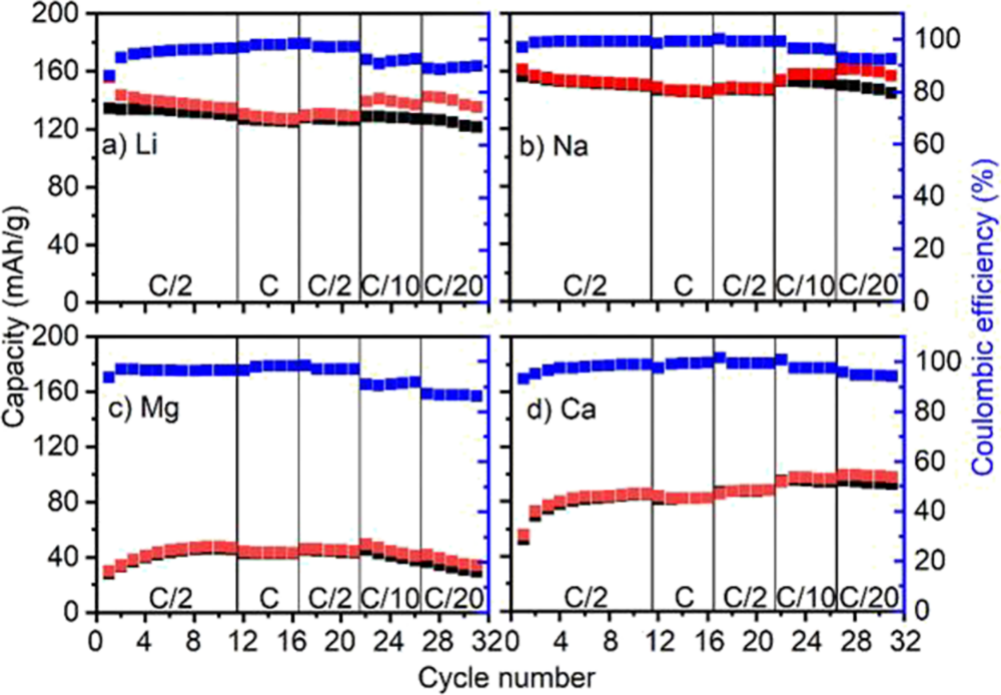
Charge (red) and discharge (black) capacities vs cycle number of PNTCDA for 1 M (a) LiTFSI and (b) NaTFSI and 0.5 M (c) Mg(TFSI)_2_ and (d) Ca(TFSI)_2_ in EC:PC with their respective Coulombic efficiencies (blue) scanned at 1C, C/2, C/10, and C/20 rates.

The electrochemical performance of the PNTCDA electrodes was assessed in Li, Na, Mg, and Ca based organic electrolytes. In Figure 2, we present the GCPL potential vs capacity profiles of PNTCDA electrodes at C-rates ranging from C/20 to 1C using (a) 1 M LiTFSI, (b) 1 M NaTFSI, (c) 0.5 M Mg(TFSI)_2_, and (d) 0.5 M Ca(TFSI)_2_ in EC:PC (1:1). In addition to the present remarkable experimental capacity and capacity retention in Li and Na electrolytes (ca. 135 and 150 mAh g^–1^, respectively, for Li^+^ and Na^+^ at C/2), PNTCDA electrodes also show promising electrochemical performances in divalent cells (90 and 45 mAh g^–1^, respectively for Ca^2+^ and Mg^2+^ at C/2). All potential-capacity curves follow a sloping plateau with charge/discharge voltage centered at about −0.5, −0.6, −0.7, and −0.8 V vs Ag_2_S, respectively, for Ca, Mg, Li, and Na cells. The voltage hysteresis between charge and discharge is rather small, about 200 mV for Li, Na, and Ca cells, being only slightly larger for Mg cells (ca. 250 mV). These voltage hystereses are only slightly affected by the C-rate resulting in excellent rate capability in all cases (Figure 3). Among all, only the Na cell clearly displays two close plateaus (Figure 2b) which according to Song et al. might indicate a two-step redox reaction involving two over four carbonyl groups (two electrons) in Li cells.^32,42^ The presence of these plateaus in GPCL traces has also been observed for PNTCDA with 1 M NaClO_4_ in EC and diethyl carbonate (1:1)^34^ and conjugated polymers incorporating PNTCDA blocks in their porous framework with 1 M LiTFSI in dioxalane and dimethoxyethane (1:1).^31^

In Figure 2, we also present CV curves with all four cations in order to better identify reaction potentials and see if they could support two-step redox reactions. The Na cell presents two distinct peaks in oxidation at −0.36 and 0.02 V vs Ag/Ag_2_S that remains at the same position upon cycling, while the Li one has a single peak at 0.08 V vs Ag/Ag_2_S that is most likely the convolution of two peaks, judging its width and asymmetry. Differential capacity vs voltage curve (Figure S5 plotted from GCPL curves in (Figure 2a) further highlights the presence of two different redox features. Interestingly, both divalent cells also have two distinct peaks, but in contrast with both monovalent cells, the peak positions and intensities evolve from a single peak into dual peaks upon cycling. For the Mg cell, the oxidation peak at −0.36 V vs Ag/Ag_2_S of the first cycle is split into a large and a small peak, respectively, at −0.09 and 0.17 V vs Ag/Ag_2_S (Figure 2g). Similarly, the Ca cell displays a single peak on its first cycle at 0.18 V vs Ag/Ag_2_S which is split into two peaks of similar intensities and area at −0.17 and 0.1 V vs Ag/Ag_2_S at cycle 30 (Figure 2h). For both divalent cells, this behavior may indicate a reorganization of the polymeric chains during the first cycles which may influence and modify the reaction potentials of the redox reaction of carbonyls.^43^ Both the presence of plateaus on GCPL traces and dual peaks on CVs may be consistent with a two-step redox reaction involving the successive formation of a radical anion and then a dianion in a similar way of quinones or polyquinones.^42,44^ Therefore, if all four cations are susceptible to occupy two over four active sites, Ca^2+^ and Mg^2+^ would either stand in between two C–O^–^ bonds of different monomers or as a monovalent cation with its associated anion (TFSI^–^ in our case) to a single C–O^–^ bound.

Another interesting observation from GCPL and CV studies is that there is a positive shift in the redox potential when changing the charge carriers from monovalent to divalent cations. Indeed, the average charge/discharge potential of −0.45, −0.6, −0.7, and −0.8 V vs Ag_2_S were recorded, respectively, for Ca, Mg, Li, and Na cells (Figures 2 and S3). Binding energies and redox potentials of PNTCDA are calculated for the formation of complexes between the NTCDA anion and different cation complexes (See Supporting Information for the detailed Computational Method section) and are collected in Table 1. As previously demonstrated by our group in the aqueous media^45,46^ and also by Abruña and co-workers^47^ in the organic electrolytes, the positive gain in the reduction potential (thermodynamic stabilization) is ascribed to the stronger binding (*E*_bind,Mg(TFSI)]_^+^ = −14.1/–19.9 vs *E*_bind,Na_^+^ = −12.5/–18.6 kcal/mol) of Mg(TFSI)^+^ over Na^+^ charge carriers with the enolates. This experimentally observed trend of redox potential gain is also corroborated by the calculated redox potential values (Table 1). It is important to mention that in the case of 0.5 M Mg(TFSI)_2_, the main charge carrier is considered to be Mg(TFSI)^+^ as reported elsewere.^41^ The redox mechanism considered for the calculations was a sequential 2e^–^ reduction in two steps as suggested by the double-redox peaks observed in CVs (Figure 2f,g).

**Table 1:** Electrochemical and Modeling Parameters for PNTCDA in an Organic Electrolyte Containing Representative Na- and Mg-Based Charge Carriers

electrolyte	charge carrier^a^	*E*_red1,exp_ (V)^b^	*E*_red2,exp_ (V)^b^	*E*_red1,calc_ (V)^c^	*E*_red2,calc_ (V)^c^	Δ*G*_bind_ (kcal/mol)^c^
1 M NaTFSI	[Na]^+^	–0.95	–0.65	4.25	4.04	–12.5/–18.6
0.5 M Mg(TFSI)_2_	[Mg(TFSI)]^+^		–0.6	4.32	4.08	–14.1/–19.9

a EC molecules from [Na(EC)_2_]^+^ and [Mg(EC)_3_(TFSI)]^+^ complex charge carriers are been omitted for simplicity.

b *E*_red1,exp_ and *E*_red2,exp_ obtained experimentally in GCPL at C/10. The potentials are referred against Ag_2_S.

c Refer to the Supporting Information to see the full details of the computational method to calculate *E*_red,calc_ and *E*_binding_. The absolute values of *E*_red,calc_ are reported here. The Δ*G*_bind_ values correspond to the binding of the first and second cation charge carriers.

Additionally, both steric effects and strong ion pairings^41,48,49^ may explain the discrepancy between monovalent and divalent capacities with 140 and 150 mAh g^–1^, respectively, for Li^+^ and Na^+^ and 90 and 45 mAh g^–1^, respectively, for Ca^2+^ and Mg^2+^ (Figure 3), with the Mg^2+^ cell having the lowest capacity as contact ion-pair formation is known to be exacerbated with Mg.^41^Figure 3 shows that both divalent cells undergo an activation step during the first five cycles at C/2 with a continuous capacity increase. While the origin of this activation period remains unclear, it can be related to electrode wetting issues and ionic path improvement over time. The first cycles at 1C exhibit Coulombic efficiencies of 86, 97, 94, and 93% for Li, Na, Mg, and Ca cells, respectively. For the subsequent 10 cycles at 1C and C/2, the Coulombic efficiency of Li^+^ and Mg^2+^ cells increased to 96%, while for Na and Ca cells, it increased to 99%. Modifying the C-rates has little impact on the capacity, but lower Coulombic efficiencies are recorded at low C-rates (C/10 and C/20), which may be originated from the parasitic polymer and/or electrolyte reactions. All cells present very good capacity retention upon cycling at C/2 and 1C, and slight (Li and Na) or relatively fast (Mg) capacity fading takes place at C/10 and C/20. By contrast, the Ca cell capacity increases when the C-rate is decreased down to C/10 and C/20. Optimistically, high rate performance of PNTCDA, particularly in Ca cells, is highly encouraging since achieving satisfactory performance in these conditions is still an issue for multivalent batteries. Upon 20-fold increase of current from C/20 to 1C, a slight decrease in specific capacity (11%) was observed in the Ca cell, yet attaining a high capacity output of 80 mAh g^–1^ at 1C. This performance is far superior when compared to the traditional inorganic and advanced organic electrodes in Ca-ion cells, except the best-performing 3,4,9,10-perylene tetracarboxylic dianhydride^50^ (Table S1).

**Figure 4:**
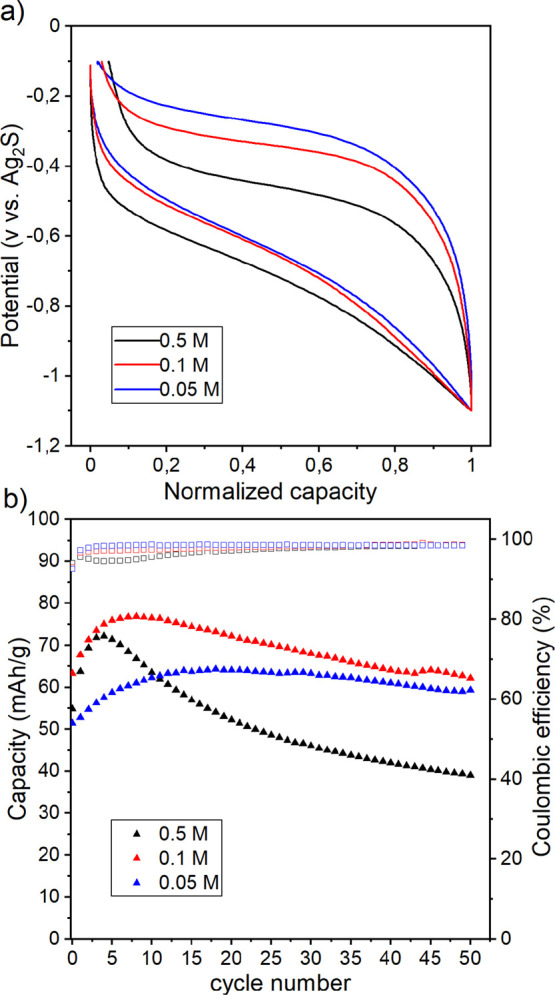
(a) Normalized voltage versus capacity profiles (third cycle) and (b) discharge capacities vs cycle number (Coulombic efficiencies in empty squares) of PNTCDA electrodes in (blue) 0.05 M, (red) 0.1 M, and (black) 0.5 M Mg(TFSI)_2_ in EC:PC.

Since Mg-based electrolytes are more prone to ion-pair formation, cycling of PNTCDA was performed using electrolytes with different Mg(TFSI)_2_ concentrations (0.5, 0.1, and 0.05 M) in order to assess the impact of ion-pairs on the electrochemical performances. It was previously demonstrated that the electrolyte with salt concentration higher than 0.1 M presents significant degree of contact ion-pair.^41^ Cells were cycled at C/2 rate for 50 cycles (Figure 4). Charge/discharge profiles in Figure 4a show that the overpotential increases when the concentration of Mg salt decreases, probably due to the lower conductivity of the more diluted Mg-based electrolytes. It is also worth noting that the average charge/discharge potential is also salt concentration-dependent with a shift toward higher values for lower concentration (less contact ion-pairs).

**Figure 5:**
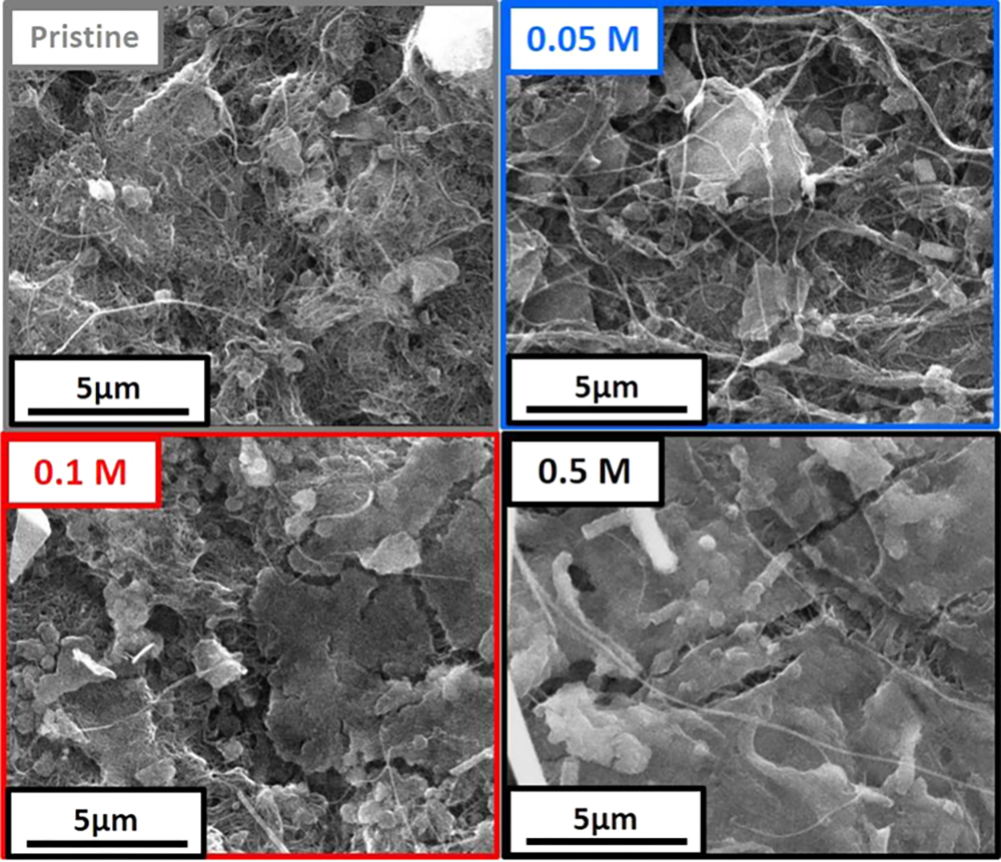
SEM images of PNTCDA electrodes as prepared (pristine) or stopped after 50 cycles (end of reduction) in 0.05, 0.1, or 0.5 M Mg(TFSI)_2_ in EC:PC.

Figure 4b shows that capacities after 50 cycles are similar for 0.1 and 0.05 M (about 60 mAh g^–1^), while at 0.5 M, a strong capacity fading upon cycling is observed and 40 mAh g^–1^ is reached. Moreover, a passive layer observed with SEM imaging is formed and appears to grow thicker and denser with the increase in the Mg concentration in the electrolyte (Figure 5). Overall, the evolution of capacity with the number of cycles follows the same trend for the three different concentrations with an initial increase of capacity followed by capacity fading. The capacity of cells with 0.5 and 0.1 M concentrations increases for about five cycles and then drops from 72 to 39 mAh g^–1^ and from 76 to 62 mAh g^–1^ that corresponds to 55 and 81% of capacity retention after 50 cycles, respectively. The 0.05 M cell tend to display the best performance in term of stability as we observe a capacity retention of 92% after the stabilization and a capacity of 60 mAh g^–1^ after 50 cycles. Moreover, the study of the Coulombic efficiency indicates that for both 0.5 and 0.1 M cells, it takes, respectively, 32 and 22 cycles to reach a Coulombic efficiency of 98%, while it takes only four cycles for the 0.05 M cell to surpass 98%. The initial capacity increase may be ascribed to electrode wettability increase and/or an initial modification of the electrode microstructure, while the loss of capacity can be ascribed to the irregular growth of the passive layer over time that affects the porosity and overall ionic transport. Therefore, decreasing the concentration clearly improves the system stability and may be also related to the decreased concentration of ion-pairs in diluted electrolytes. Indeed, a high proportion of ion-pairs may favor the formation of this passive layer as it would bring TFSI anions as [Mg(TFSI)]^+^ complexes in larger quantities at the electrode surface. Moreover, a high proportion of ion-pairs may also yield the coordination of Mg^2+^ and TFSI^–^ species in the electrode structure that would negatively affect the accessibility of the carbonyl sites, and together with the passive layer formation, it would explain why cells with a divalent cation, particularly Mg^2+^, have lower capacities than monovalent cation-based cells (Figure 3). Finally, the formation of ion-pairs implies active cation with lower charge density and, as discussed previously, should result in lower operation potential of the PNTCDA electrode. The fact that the operation potential increases from about −0.5 to −0.4 V vs Ag/Ag_2_S when the salt concentration is decreased from 0.5 to 0.05 M (Figure 4a) also indicates that the operation potential can be tuned depending on the solvation structure of a given cation, with fully dissociated salt promoting high operation potential. Such a modified operation potential depending on the solvation structure of cations was highlighted previously by Ernould et al. and constitutes a promising strategy to raise the cell voltage and thus the energy density.^51^

## Conclusions

A comparative study has been carried out on polyimide cathodes in Li, Na, Mg, and Ca cells. Operando FTIR spectroscopy allowed assessing the reversible mechanism for the conversion of redox-active carbonyl groups via enolation/carbonylation reactions. GCPL traces and CVs are consistent with a two-step redox reaction involving the successive formation of a radical anion and then a dianion as suggested previously for quinones or polyquinones. Remarkable capacity and capacity retention in Li and Na based electrolytes (>135 mAh g^–1^ at C/2) and promising electrochemical performances in divalent cells (90 and 45 mAh g^–1^, respectively for Ca^2+^ and Mg^2+^ at C/2) were recorded.

The larger divalent cation solvation shell due to higher coordination number and higher tendency to form ion-pairs may explain the discrepancy between monovalent and divalent capacities, with Mg cell having the lowest capacity as contact ion-pair formation is known to be exacerbated compared with other cations. Such a difference in the cation solvation structure also results in more significant electrode swelling and an initial capacity increase during the first few cycles (activation period) with divalent cation-based electrolytes. While the origin of this activation period remains unclear and requires further investigation, we believe it can be related to electrode wetting issues and ionic path improvement over time, with divalent cation complexes (larger than Li and Na ones) requiring more important polymer structural evolution in order to facilitate the access to the redox centers. Therefore, it is expected that careful cation solvation shell engineering, aiming at more compact cation complexes, could be beneficial in terms of material utilization (specific capacity).

All cells present rather small voltage hysteresis being only slightly affected by the C-rate, resulting in excellent rate capability as well as good Coulombic efficiencies with 96% for Li^+^ and Mg^2+^ cells and 99% for Na and Ca. Such performances are highly encouraging, especially for the development of Ca and Mg based batteries for which achieving satisfactory performance is still extremely challenging. In particular, these performances are far superior when compared to the traditional inorganic and advanced organic electrode in Ca-ion cells.

A positive shift in the redox potential was observed with increasing strength of the cation-enolate binding, and therefore moving from monovalent to divalent cations can result in a positive gain in terms of battery output voltage and thus energy density. However, such a benefit is hindered by ion-pair formation as it limits the cation-enolate binding strength. This is particularly detrimental for Mg-based electrolytes because of the high charge density of Mg^2+^. Furthermore, the salt concentration significantly impacts the average charge/discharge potential as lowering the salt concentration favorably limits the ion-pair formation. Most importantly, decreasing the salt concentration in Mg cells also greatly improves the cyclability, with a high degree of ion-pairs favoring anion decomposition and passivation layer formation.

Overall, this work highlights various promising strategies for the development of Ca and Mg organic-based cathodes with improved capacity, cycle life, and operating potential.

## Supporting Information Available

Operando FTIR spectra of PNTCDA electrodes, PNTCDA buckypaper electrode preparation, computational methods, computational results, and comparison of electrochemical performance of PNTCDA in Ca^2+^ electrolyte with previously reported Ca-organic cells (PDF)

## References

[ref1] Yang Y.; Okonkwo E. G.; Huang G.; Xu S.; Sun W.; He Y. On the sustainability of lithium ion battery industry – A review and perspective. Energy Storage Mater. 2021, 36, 186–212. 10.1016/j.ensm.2020.12.019.

[ref2] Gruber P. W.; Medina P. A.; Keoleian G. A.; Kesler S. E.; Everson M. P.; Wallington T. J. Global Lithium Availability. A Constraint for Electric Vehicles?. J. Ind. Ecol. 2011, 15, 760–775. 10.1111/j.1530-9290.2011.00359.x.

[ref3] Zeng X.; Yang C.; Chiang J. F.; Li J. Innovating e-waste management: From macroscopic to microscopic scales. Sci. Total Environ. 2017, 575, 1–5. 10.1016/j.scitotenv.2016.09.078. 27723459

[ref4] Chen M.; Ogunseitan O. A.; Wang J.; Chen H.; Wang B.; Chen S. Evolution of electronic waste toxicity: Trends in innovation and regulation. Environ. Int. 2016, 89-90, 147–154. 10.1016/j.envint.2016.01.022. 26854858

[ref5] Yabuuchi N.; Kubota K.; Dahbi M.; Komaba S. Research Development on Sodium-Ion Batteries. Chem. Rev. 2014, 114, 11636–11682. 10.1021/cr500192f. 25390643

[ref6] Vaalma C.; Buchholz D.; Weil M.; Passerini S. A cost and resource analysis of sodium-ion batteries. Nat. Rev. Mater. 2018, 3, 18013 10.1038/natrevmats.2018.13.

[ref7] Arroyo-de Dompablo M. E.; Ponrouch A.; Johansson P.; Palacín M. R. Achievements, Challenges, and Prospects of Calcium Batteries. Chem. Rev. 2020, 120, 6331–6357. 10.1021/acs.chemrev.9b00339. 31661250

[ref8] Stievano L.; de Meatza I.; Bitenc J.; Cavallo C.; Brutti S.; Navarra M. A. Emerging calcium batteries. J. Power Sources 2021, 482, 228875 10.1016/j.jpowsour.2020.228875.

[ref9] Zhao-Karger Z.; Fichtner M. Beyond Intercalation Chemistry for Rechargeable Mg Batteries: A Short Review and Perspective. Front. Chem. 2019, 6, 656 10.3389/fchem.2018.00656. 30697538 PMC6341060

[ref10] Mineral Commodity Summaries 2021; U.S. Geological Survey, 2021.

[ref11] Monti D.; Ponrouch A.; Araujo R. B.; Barde F.; Johansson P.; Palacín M. R. Multivalent Batteries—Prospects for High Energy Density: Ca Batteries. Front. Chem. 2019, 7, 79 10.3389/fchem.2019.00079. 30842941 PMC6391315

[ref12] Muldoon J.; Bucur C. B.; Gregory T. Fervent Hype behind Magnesium Batteries: An Open Call to Synthetic Chemists—Electrolytes and Cathodes Needed. Angew. Chem., Int. Ed. 2017, 56, 12064–12084. 10.1002/anie.201700673. 28295967

[ref13] Goujon N.; Casado N.; Patil N.; Marcilla R.; Mecerreyes D. Organic batteries based on just redox polymers. Prog. Polym. Sci. 2021, 122, 101449 10.1016/j.progpolymsci.2021.101449.

[ref14] Lyu H.; Sun X.-G.; Dai S. Organic Cathode Materials for Lithium-Ion Batteries: Past, Present, and Future. Adv. Energy Sustain. Res. 2021, 2, 2000044 10.1002/aesr.202000044.

[ref15] Saal A.; Hagemann T.; Schubert U. S. Polymers for Battery Applications—Active Materials, Membranes, and Binders. Adv. Energy Mater. 2021, 11, 2001984 10.1002/aenm.202001984.

[ref16] Bitenc J.; Vizintin A.; Grdadolnik J.; Dominko R. Tracking electrochemical reactions inside organic electrodes by operando IR spectroscopy. Energy Storage Mater. 2019, 21, 347–353. 10.1016/j.ensm.2019.05.038.

[ref17] Poizot P.; Gaubicher J.; Renault S.; Dubois L.; Liang Y.; Yao Y. Opportunities and Challenges for Organic Electrodes in Electrochemical Energy Storage. Chem. Rev. 2020, 120, 6490–6557. 10.1021/acs.chemrev.9b00482. 32207919

[ref18] Passiniemi P.; Österholm J.-E. Critical aspects of organic polymer batteries. Synth. Met. 1987, 18, 637–644. 10.1016/0379-6779(87)90953-2.

[ref19] Panero S.; Prosperi P.; Bonino F.; Scrosati B.; Corradini A.; Mastragostino M. Characteristics of electrochemically synthesized polymer electrodes in lithium cells—III Polypyrrole. Electrochim. Acta 1987, 32, 1007–1011. 10.1016/0013-4686(87)90025-9.

[ref20] Yoshino A. The Birth of the Lithium-Ion Battery. Angew. Chem., Int. Ed. 2012, 51, 5798–5800. 10.1002/anie.201105006. 22374647

[ref21] MacInnes D.; Druy M. A.; Nigrey P. J.; Nairns D. P.; MacDiarmid A. G.; Heeger A. J. Organic batteries: reversible n- and p- type electrochemical doping of polyacetylene, (CH)_x_. J. Chem. Soc. Chem. Commun. 1981, 317–319. 10.1039/c39810000317.

[ref22] Wang P.-C.; Liu L.-H.; Alemu Mengistie D.; Li K.-H.; Wen B.-J.; Liu T.-S.; Chu C.-W. Transparent electrodes based on conducting polymers for display applications. Displays 2013, 34, 301–314. 10.1016/j.displa.2013.05.003.

[ref23] Liu J.; Wang M.; Xu N.; Qian T.; Yan C. Progress and perspective of organosulfur polymers as cathode materials for advanced lithium-sulfur batteries. Energy Storage Mater. 2018, 15, 53–64. 10.1016/j.ensm.2018.03.017.

[ref24] Wang S.; Li F.; Easley A. D.; Lutkenhaus J. L. Real-time insight into the doping mechanism of redox-active organic radical polymers. Nat. Mater. 2019, 18, 69–75. 10.1038/s41563-018-0215-1. 30478451

[ref25] Nishide H.; Iwasa S.; Pu Y.-J.; Suga T.; Nakahara K.; Satoh M. Organic radical battery: nitroxide polymers as a cathode-active material. Electrochim. Acta 2004, 50, 827–831. 10.1016/j.electacta.2004.02.052.

[ref26] Peng C.; Ning G.-H.; Su J.; Zhong G.; Tang W.; Tian B.; Su C.; Yu D.; Zu L.; Yang J.; Ng M.-F.; Hu Y.-S.; Yang Y.; Armand M.; Loh K. P. Reversible multi-electron redox chemistry of π-conjugated N-containing heteroaromatic molecule-based organic cathodes. Nat. Energy 2017, 2, 17074 10.1038/nenergy.2017.74.

[ref27] Lu Y.; Hou X.; Miao L.; Li L.; Shi R.; Liu L.; Chen J. Cyclohexanehexone with Ultrahigh Capacity as Cathode Materials for Lithium-Ion Batteries. Angew. Chem., Int. Ed. 2019, 58, 7020–7024. 10.1002/anie.201902185. 30916877

[ref28] Häupler B.; Wild A.; Schubert U. S. Carbonyls: Powerful Organic Materials for Secondary Batteries. Adv. Energy Mater. 2015, 5, 1402034 10.1002/aenm.201402034.

[ref29] Dong X.; Yu H.; Ma Y.; Bao J. L.; Truhlar D. G.; Wang Y.; Xia Y. All-Organic Rechargeable Battery with Reversibility Supported by “Water-in-Salt” Electrolyte. Chem. Eur. J. 2017, 23, 2560–2565. 10.1002/chem.201700063. 28075043

[ref30] Zhao Q.; Whittaker A. K.; Zhao X. S. Polymer Electrode Materials for Sodium-ion Batteries. Materials (Basel) 2018, 11, 2567 10.3390/ma11122567. 30562972 PMC6315866

[ref31] Lei S.; Dong Y.; Dou Y.; Zhang X.; Zhang Q.; Yang Y. Polymerization-tailored polyimides as cathodes for lithium-ion batteries. Mater. Adv. 2021, 2, 5785–5790. 10.1039/D1MA00554E.

[ref32] Song Z.; Zhan H.; Zhou Y. Polyimides: promising energy-storage materials. Angew. Chem., Int. Ed. 2010, 49, 8444–8448. 10.1002/anie.201002439. 20862664

[ref33] Han X.; Chang C.; Yuan L.; Sun T.; Sun J. Aromatic Carbonyl Derivative Polymers as High-Performance Li-Ion Storage Materials. Adv. Mater. 2007, 19, 1616–1621. 10.1002/adma.200602584.

[ref34] Chen L.; Li W.; Wang Y.; Wang C.; Xia Y. Polyimide as anode electrode material for rechargeable sodium batteries. RSC Adv. 2014, 4, 25369–25373. 10.1039/C4RA03473B.

[ref35] Hernández G.; Casado N.; Coste R.; Shanmukaraj D.; Rubatat L.; Armand M.; Mecerreyes D. Redox-active polyimide–polyether block copolymers as electrode materials for lithium batteries. RSC Adv. 2015, 5, 17096–17103. 10.1039/C4RA15976D.

[ref36] Xiong P.; Yin H.; Chen Z.; Zhao C.; Yang J.; Huang S.; Xu Y. Flexible polytriphenylamine-based cathodes with reinforced energy-storage capacity for high-performance sodium-ion batteries. Sci. China Mater. 2020, 63, 1929–1938. 10.1007/s40843-020-1375-2.

[ref37] Luo Z.; Liu L.; Ning J.; Lei K.; Lu Y.; Li F.; Chen J. A Microporous Covalent–Organic Framework with Abundant Accessible Carbonyl Groups for Lithium-Ion Batteries. Angew. Chem., Int. Ed. 2018, 57, 9443–9446. 10.1002/anie.201805540. 29863784

[ref38] Gao H.; Tian B.; Yang H.; Neale A. R.; Little M. A.; Sprick R. S.; Hardwick L. J.; Cooper A. I. Crosslinked Polyimide and Reduced Graphene Oxide Composites as Long Cycle Life Positive Electrode for Lithium-Ion Cells. ChemSusChem 2020, 13, 5571–5579. 10.1002/cssc.202001389. 32725860 PMC7693101

[ref39] Dugas R.; Forero-Saboya J. D.; Ponrouch A. Methods and Protocols for Reliable Electrochemical Testing in Post-Li Batteries (Na, K, Mg, and Ca). Chem. Mater. 2019, 31, 8613–8628. 10.1021/acs.chemmater.9b02776. 31736535 PMC6854841

[ref40] Fortunato B.; Mirone P.; Fini G. Infrared and Raman spectra and vibrational assignment of ethylene carbonate. Spectrochim. Acta A 1971, 27, 1917–1927. 10.1016/0584-8539(71)80245-3.

[ref41] Forero-Saboya J. D.; Marchante E.; Araujo R. B.; Monti D.; Johansson P.; Ponrouch A. Cation Solvation and Physicochemical Properties of Ca Battery Electrolytes. J. Phys. Chem. C 2019, 123, 29524–29532. 10.1021/acs.jpcc.9b07308. PMC6961307 31956392

[ref42] Song Z.; Zhan H.; Zhou Y. Anthraquinone based polymer as high performance cathode material for rechargeable lithium batteries. Chem. Commun. 2009, 5, 448–450. 10.1039/B814515F. 19137181

[ref43] Fan X.; Wang F.; Ji X.; Wang R.; Gao T.; Hou S.; Chen J.; Deng T.; Li X.; Chen L.; Luo C.; Wang L.; Wang C. A Universal Organic Cathode for Ultrafast Lithium and Multivalent Metal Batteries. Angew. Chem., Int. Ed. 2018, 57, 7146–7150. 10.1002/anie.201803703. 29704298

[ref44] Deng W.; Liang X.; Wu X.; Qian J.; Cao Y.; Ai X.; Feng J.; Yang H. A low cost, all-organic Na-ion Battery Based on Polymeric Cathode and Anode. Sci. Rep. 2013, 3, 2671 10.1038/srep02671. 24036973 PMC3773616

[ref45] Patil N.; Mavrandonakis A.; Jérôme C.; Detrembleur C.; Casado N.; Mecerreyes D.; Palma J.; Marcilla R. High-performance all-organic aqueous batteries based on a poly(imide) anode and poly(catechol) cathode. J. Mater. Chem. A 2021, 9, 505–514. 10.1039/D0TA09404H.

[ref46] Patil N.; Mavrandonakis A.; Jérôme C.; Detrembleur C.; Palma J.; Marcilla R. Polymers Bearing Catechol Pendants as Universal Hosts for Aqueous Rechargeable H^+^, Li-Ion, and Post-Li-ion (Mono-, Di-, and Trivalent) Batteries. ACS Appl. Energy Mater. 2019, 2, 3035–3041. 10.1021/acsaem.9b00443.

[ref47] Hernández-Burgos K.; Rodríguez-Calero G. G.; Zhou W.; Burkhardt S. E.; Abruña H. D. Increasing the Gravimetric Energy Density of Organic Based Secondary Battery Cathodes Using Small Radius Cations (Li^+^ and Mg^2+^). J. Am. Chem. Soc. 2013, 135, 14532–14535. 10.1021/ja407273c. 24040955

[ref48] Tchitchekova D. S.; Monti D.; Johansson P.; Bardé F.; Randon-Vitanova A.; Palacín M. R.; Ponrouch A. On the Reliability of Half-Cell Tests for Monovalent (Li^+^, Na^+^) and Divalent (Mg^2+^, Ca^2+^) Cation Based Batteries. J. Electrochem. Soc. 2017, 164, A1384–A1392. 10.1149/2.0411707jes.

[ref49] Samuel D.; Steinhauser C.; Smith J. G.; Kaufman A.; Radin M. D.; Naruse J.; Hiramatsu H.; Siegel D. J. Ion Pairing and Diffusion in Magnesium Electrolytes Based on Magnesium Borohydride. ACS Appl. Mater. Interfaces 2017, 9, 43755–43766. 10.1021/acsami.7b15547. 29134805

[ref50] Chae M. S.; Nimkar A.; Shpigel N.; Gofer Y.; Aurbach D. High Performance Aqueous and Nonaqueous Ca-Ion Cathodes Based on Fused-Ring Aromatic Carbonyl Compounds. ACS Energy Lett. 2021, 6, 2659–2665. 10.1021/acsenergylett.1c01010.

[ref51] Ernould B.; Sieuw L.; Barozzino-Consiglio G.; Gohy J.-F.; Vlad A. Negative Redox Potential Shift in Fire-Retardant Electrolytes and Consequences for High-Energy Hybrid Batteries. ACS Appl. Energy Mater. 2019, 2, 7879–7885. 10.1021/acsaem.9b01339.

