# Supplementary material for: Polyimides as Promising Cathodes for Metal–Organic Batteries: A Comparison between Divalent (Ca2+, Mg2+) and Monovalent (Li+, Na+) Cations
Source: ACS Appl Energy Mater. 2023 Jun 27;6(13):7250–7. doi: 10.1021/acsaem.3c00969 (PMC10336839; doi:10.1021/acsaem.3c00969)
Supplement: Supplementary file 1 — ae3c00969_si_001.pdf [file ae3c00969_si_001.pdf]

Supporting Information

Polyimides as Promising Cathodes for Metal Organic Batteries: A Comparison Between Divalent (Ca^{2+}, Mg^{2+}) and Monovalent (Li^{+}, Na^{+}) Cations

Damien Monti[1], Nagaraj Patil [2], Ashley P. Black[1], Dionysios Raptis[6], Andreas Mavrandonakis[2], George E. Froudakis[6], Ibraheem Yousef[3], Nicolas Goujon[4,7], David Mecerreyes[4], Rebeca Marcilla[2]*, Alexandre Ponrouch[1,5]*

[1] Institut de Ciència de Materials de Barcelona (ICMAB-CSIC), Campus UAB, 08193 Bellaterra, Catalonia (Spain)

[2] Electrochemical Processes Unit, IMDEA Energy, Avda. Ramón de La Sagra 3, 28935 Móstoles (Spain).

[3] MIRAS Beamline, ALBA Synchrotron Light Source, Carrer de la Llum 2-26, 08290 Cerdanyola del Vallès (Spain)

[4] POLYMAT University of the Basque Country UPV/EHUAvenida Tolosa 72, Donostia-San Sebastián 20018 (Spain)

[5] ALISTORE − European Research Institute, CNRS FR 3104, Hub de l'Energie, 15 Rue Baudelocque, 80039 Amiens (France)

[6] Department of Chemistry, University of Crete, Voutes Campus, GR-71003 Heraklion, Crete, Greece

[7] Centre for Cooperative Research on Alternative Energies (CIC energiGUNE), Basque Research and Technology Alliance (BRTA), Alava Technology Park, Albert Einstein 48, 01510, Vitoria-Gasteiz, Spain.

Corresponding authors*: Rebeca Marcilla (rebeca.marcilla@imdea.org) and Alexandre Ponrouch (aponrouch@icmab.es)

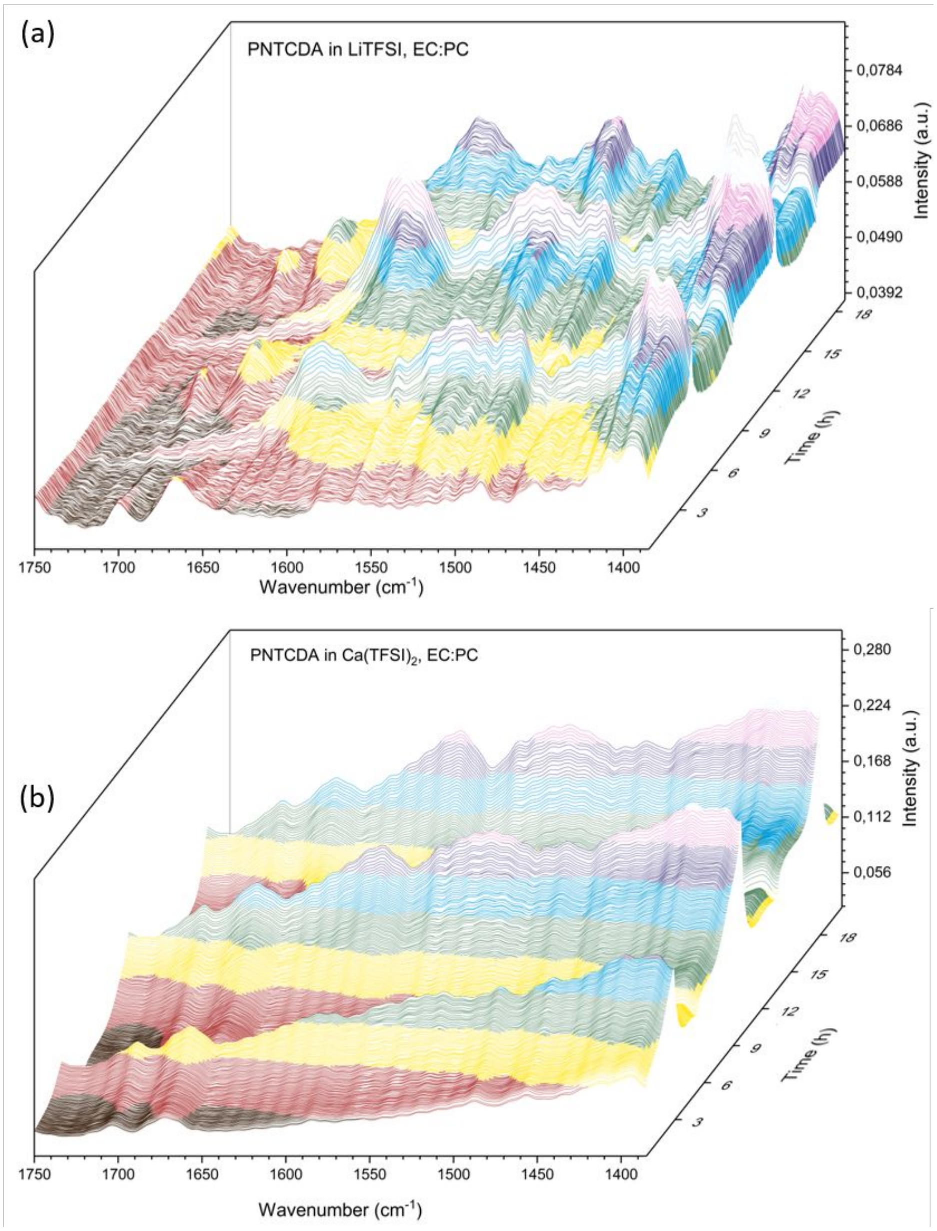

Figure S1. Operando Fourier Transform infrared spectroscopy spectra of PNTCDA electrodes cycled in 1M LiTFSI, EC:PC (a) and in 0.5M $Ca(TFSI)_2$, EC:PC (b).

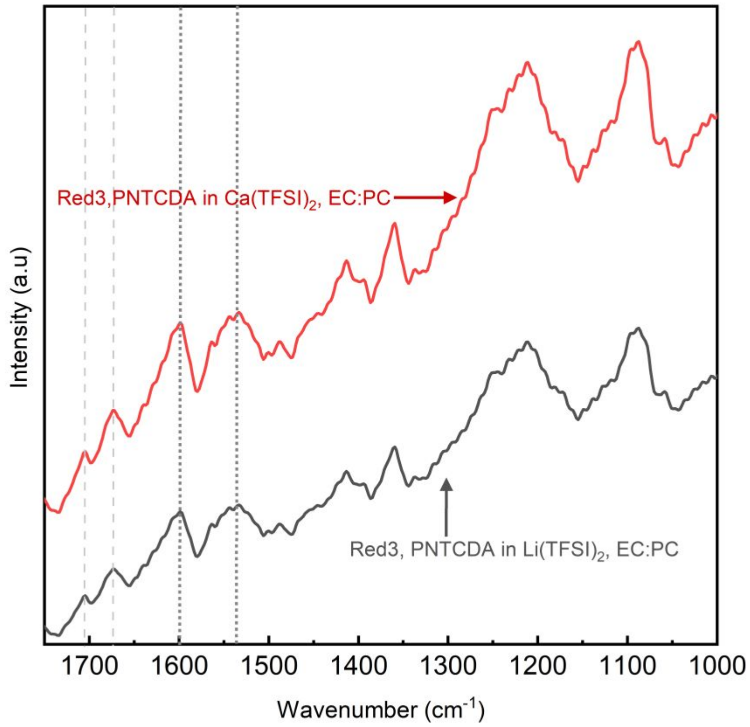

Figure S2. Comparison of FTIR spectra of PNTCDA electrodes cycled in Li (grey curve) and Ca electrolytes (red curve) at the end of the third reduction.

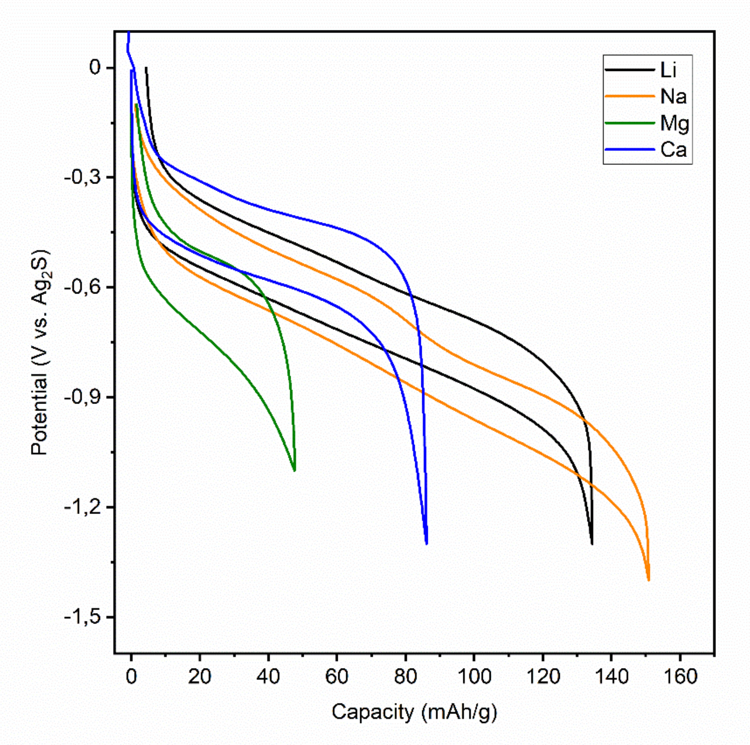

Figure S3. Comparison GCPL potential (vs. Ag/Ag_2S) vs. capacity curves of PNTCDA in 1 M (a) LiTFSI, (b) NaTFSI and 0.5 M (c) $Mg(TFSI)_2$ (d) $Ca(TFSI)_2$ in EC:PC for the last cycles at C/2 rate

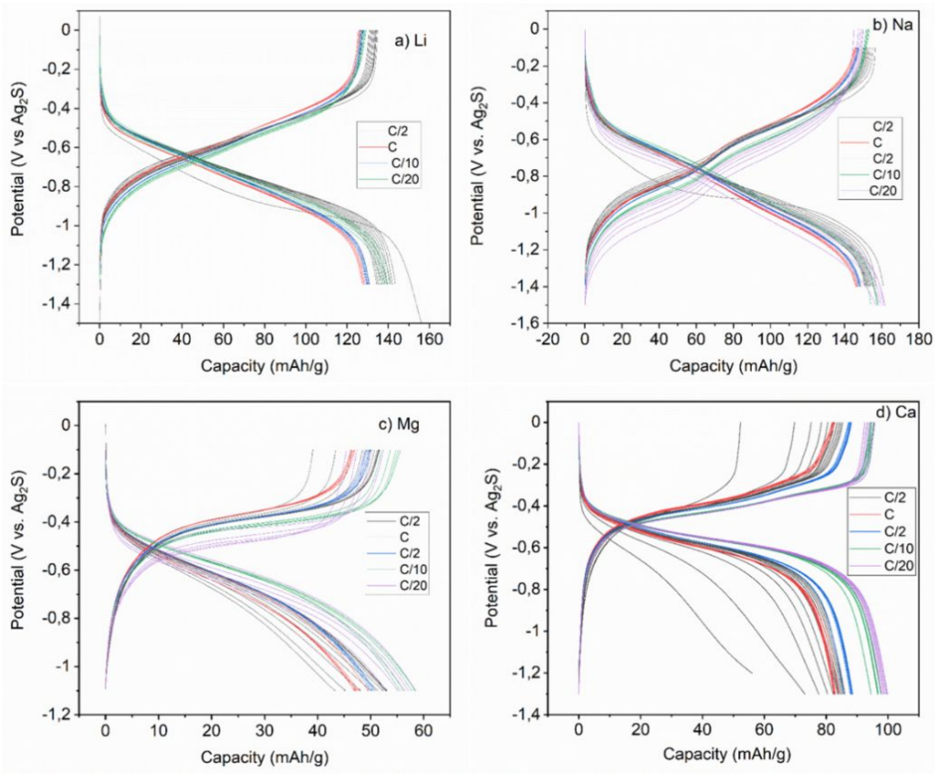

Figure S4. GCPL potential (*vs.* Ag/Ag_2S) vs. capacity curves of PNTCDA in 1 M (a) LiTFSI, (b) NaTFSI and 0.5 M (c) $Mg(TFSI)_2$ (d) $Ca(TFSI)_2$ in EC:PC, cycling at different C rates corresponding to the capacity vs. cycle number in figure 3.

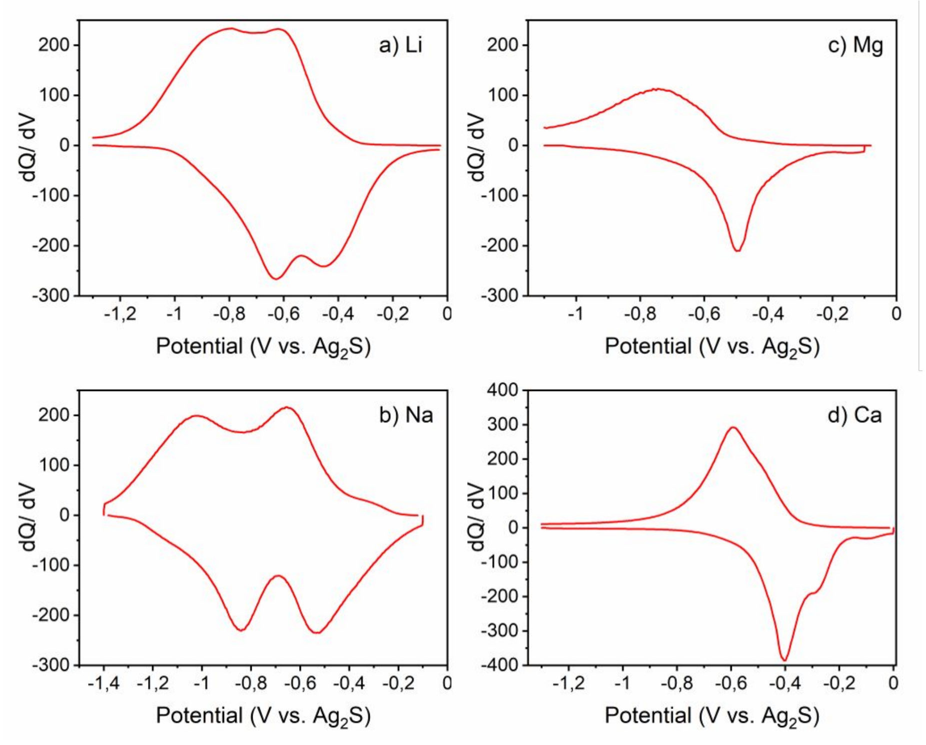

Figure S5. Differential capacity *vs*. voltage (dQ/dV) curves for PNTCDA in 1 M (a) LiTFSI, (b) NaTFSI and 0.5 M (c) $Mg(TFSI)_2$ (d) $Ca(TFSI)_2$ in EC:PC at 1C obtained from GCPL curves in Figure 2a, 2b, 2c and 2d.

PNTCDA buckypaper electrode preparation: The self-standing, binder- and metal current collector-free buckypaper electrodes were prepared following the modified procedure previously reported by us[1]. In brief, first, 8.3 mg of single-walled carbon nanotubes (SWCNTs) are dispersed in 20 mL solution of *iso*-propanol (IPA)/N-methyl-2-pyrrolidone (NMP) (1/1 v/v) through a tip sonicator for 10 min, full power, three-quarter amplitude (ultrasonic processor UP400S, 400 W, 24 kHz). Then, after carefully grinding 8.3 mg of reduced graphene oxide (RGO) with 25 mg of PNTCDA, those are added to the SWCNTs dispersion and then sonicated again at half power, half amplitude for another 10 min. After, this dispersion is immersed in a bath sonicator (Branson 2510, 100 W, 42 kHz) for 2 h, then stirred overnight to prepare the electrode ink. The next step is the filtration of the electrode ink. The suspension was filtered through a Nylon membrane filter (47 mm diameter, pore size 0.45 um) with the help of vacuum, followed by thorough rinsing with IPA to remove loosely bound polymer. The buckypaper was carefully peeled-off from the filter and dried overnight at 60 °C under vacuum. The buckypaper was cut into circular discs with a diameter of 10 mm with an average PNTCDA mass loading of 2.5 mg cm^{-2}.

Computational methods:

The structure of 1,4,5,8-naphthalenetetracarboxylic dianhydride (NTCDA)-derived polyimide (PNTCDA) is truncated to a dimer unit that is composed by two naphthalenetetracarboxylic diimide (denoted as NTCDA) units terminated with a $–CH_3$ group at the nitrogen position. The geometries of all structures are optimized with the r2-SCAN-3c density functional and the def2-mTZVPP basis set for all atoms. The structures are verified to be minima after performing a frequency analysis, where no imaginary frequencies are computed. A final electronic energy calculation is carried out in liquid phase with the solvent treated by the implicit universal solvation model SMD. Since no parameters exist for a 1:1 mixture of propylene carbonate (PC) and ethylene carbonate (EC), we decided to use the mean values of the two carbonate solvents. Free energies are calculated assuming T = 298.15 K and P = 1 atm, based on the quasi-harmonic oscillator approximation from the vibrational partition functions. The low-lying vibrational modes below 50 cm^{-1} are substituted with 50 cm^{-1} during the analysis of the thermodynamic properties. The calculation of thermodynamic properties (Gibbs free energies) are performed with the AaronTools package[2]. All DFT calculations have been performed utilizing the ORCA 5.0.2 package[3].

Binding energies and redox potentials of the PNTCDA are calculated for the formation of complexes between the NTCDA anion and Na^+ and Mg^{2+} metal cations that are considered to have their first coordination sphere filled with solvent molecules. For the case of magnesium, we consider that a TSFI anion is attached to the coordination sphere, thus it is modelled as $[Mg^{2+}(EC)_5(TFSI)]^{1+}$. Since the combination of including a mixture of PC and EC in the coordination sphere of the metal cations will create a large number of possible structures, we simplify by considering only EC molecules in the coordination sphere. Initially, many possible conformers of NTCDA-$M_x(EC)_y$ are generated with the crest tool, which performs calculations with the *xtb* semi-empirical code based on the GFN2-xTB formalism[4]. Subsequently, the conformers are energetically sorted by using the *censo* algorithm[5], which optimizes the gas-phase geometries with the r2-SCAN-3c method. The ten most stable conformers are further optimized using tighter criteria (TIGHTSCF, TIGHTOPT and DEFGRID3 keywords) and the free energies in solution are computed. The calculation of the total binding energies of the bireduced $NTCDA^{2-}$ with the metal cations is done with the following equation: $NTCDA^{2-} + 2[M(EC)_x]^{1+} \rightarrow [NTCDA - M_2(EC)_{2x-2z}] + 2z(EC)$. The calculation of the binding energy of the first metal cation with the monoreduced $NTCDA^-$ is done from the following equation: $NTCDA^{1-} + [M(EC)_x]^{1+} \rightarrow [NTCDA - M(EC)_{x-z}] + z(EC)$, where x is the number of solvent molecules in the coordination sphere of each cation, with $x(Na^+)=4$, and $x(Mg^{2+})=6$, and M^+= Na, Mg(TFSI). Similarly, the first and second redox potentials are calculated from the following equations: $NTCDA^0 + [M(EC)_x]^{1+} + 1e^- \rightarrow [NTCDA - M(EC)_{x-z}] + z(EC)$, and $\rightarrow [NTCDA - M(EC)_{x-z}] + [M(EC)_x]^{1+} + 1e^- \rightarrow [NTCDA - M_2(EC)_{2x-2z}] + z(EC)$. The binding energies and redox potentials are calculated in the same way as in our previous works, where catecholate- and imide-based polymers were studied[6, 7]. The effect of the different concentrations of magnesium salt is considered by adding the term RTln$\frac{Q^{0'}}{Q^0}$ to the reduction free energies, where $Q^{0'}$ is the desired concentration (e.g. 0.5M), while the standard-state concentrations Q^0 are assumed to be 1 M.

Computational Results:

Initially, we want to compare the results obtained with the r2-SCAN-3c/def2-mTZVPP with the M06/6-31+G(d,p) method, which has been used in an earlier work to study the binding of Na^+ and Mg^{2+} cations with a monomeric $NTCDA$ unit[8]. In that work, the calculations were carried out in

aqueous phase with the solvent treated by the implicit universal solvation model SMD. The values obtained here with the r2-SCAN-3c/def2-mTZVPP method are -35.5 (Na^+) and -31.2 (Mg^{2+}) kcal/mol, which are very close to the M06/6-31+G(d,p) values of -29.9 (Na^+) and -30.1 (Mg^{2+}) kcal/mol. Therefore, the r2-SCAN-3c/def2-mTZVPP method is accurate enough for the systems studied here, considering the significant increase in the size of the models compared to previous works, where the polymer was simulated by one monomeric unit[7, 8].

Subsequently, the sequential $2e^-$ reduction in 2 steps is studied for Na^+ and Mg^{2+}. In the case of magnesium, the charge carrier is considered to be the $[Mg^{2+}(EC)_5(TFSI)^-]^+$ species. The results are shown in Table 1 (main manuscript). The molecular models of the most important species considered in the calculations are presented in Figure S4.

The computed first and second reduction potentials are well correlated with the binding energies of the first and second metal carrier. Stronger binding (more negative value) of the metal to the $NTCDA^{-1/-2}$ causes the first/second reduction potentials to shift to higher values.

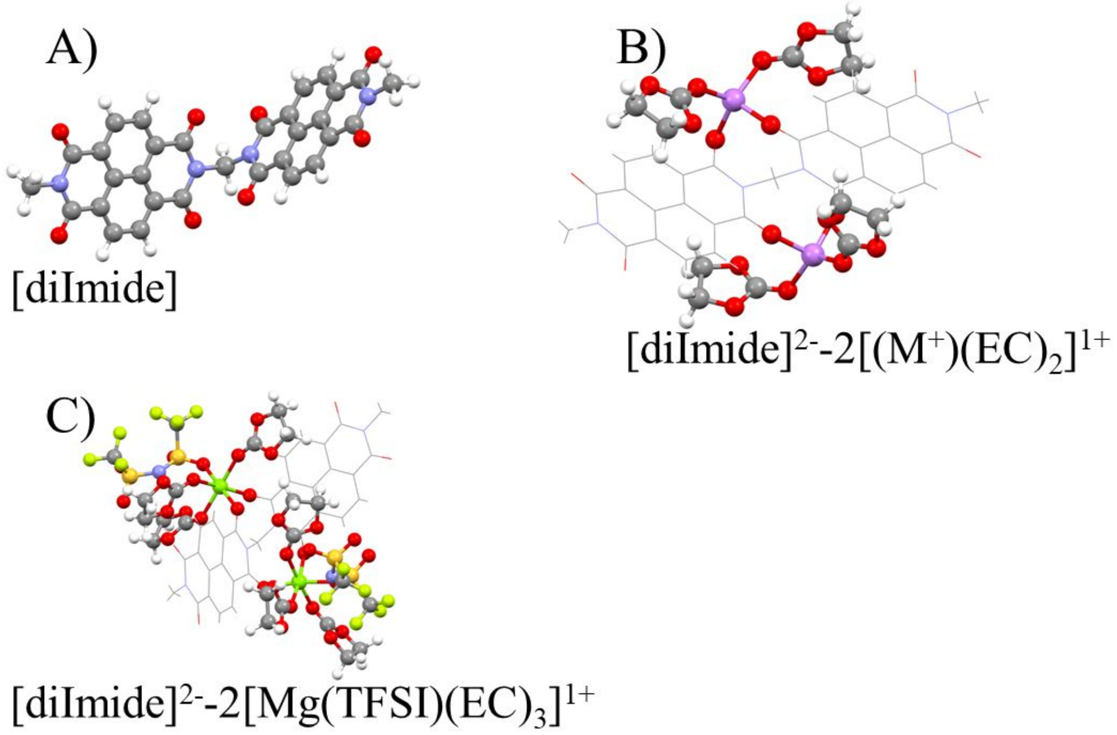

Figure S4. *Optimized structures of the A) diimide unit, B) diimide reduced by two $M^+(EC)_2$ monovalent charge carriers, with M^+=Li, Na, and C) diimide reduced by two $[Mg^{2+}(TFSI)(EC)_3]^{1+}$ monovalent charge carriers.*

Table S1: Comparison of electrochemical performance of *PNTCDA* in Ca^{2+} electrolyte with previously reported Ca-organic cells.

Sl No	Cell configuration (anode/cathode)	Electrolyte	Specific capacity	Specific capacity at the end of cycling; Current	Reference
**1**	**AC/PNTCDA**	**0.5 M $Ca(TFSI)_2$ in EC:PC**	**90 mAh g^{-1} @ 18.4 mA g^{-1} (C/20)**<br>**87 @ C/2**<br>**80 @ 1C**	**90 mAh g^{-1} @ C/20 after 32 cycles**	**This work**
2	3,4,9,10-perylene tetracarboxylic dianhydride/Graphite	3.5 m $Ca(FSI)_2$ in EC/PC/DMC/EMC	75.4 @ 100 mA g^{-1}<br>40 @ 300 mA g^{-1}	75 mA g^{-1} @ 100 mA g^{-1} after 350 cycles	9
3	AC/3,4,9,10-perylene tetracarboxylic dianhydride	Sat. $Ca(ClO_4)_2$ in PC	158 @ 10 mA g^{-1}<br>153 @ 250 mA g^{-1}	100 mA g^{-1} @ 1 A g^{-1} after 500 Cycles	10
4	Ca/PAQS	$Ca[B(hfip)_4]_2{\cdot}4dme$ in DME	110 @ C/2	114 mA g^{-1} @ C/2 after 10 Cycles	11
5	AC/$KNiFe(CN)_6$	0.5 m $Ca(TFSI)_2$ in ACN	45 @ 25 μA cm^{-2}	40 mA g^{-1} @ 25 μA cm^{-2} after 12 Cycles	12
6	BP2000 /$NiFe(CN)_6$	0.2 m $Ca(PF_6)_2$ in EC:PC	60 @ 10 mA g^{-1}	20 mA g^{-1} @ 10 mA g^{-1} after 50 Cycles	13
7	ACC/ $VOPO_4{\cdot}2H_2O$	0.8 m $Ca(TFSI)_2$ in EC:PC:EMC:DMC	100 @ 20 mA g^{-1}	45 mA g^{-1} @100 mA g^{-1} after 200 Cycles	14
8	AC/N_1VPF_3	0.25 m $Ca(PF_6)_2$ EC/PC/DMC/EMC	110 @ 10 mA g^{-1}<br>85 @ 200 mA g^{-1}	55 mA g^{-1} @250 mA g^{-1} after 2000 Cycles	15

Supplementary References.

1. A. Molina, N. Patil, E. Ventosa, M. Liras, J. Palma and R. Marcilla, *ACS Energy Letters*, 2020, **5**, 2945-2953.
2. V. M. Ingman, A. J. Schaefer, L. R. Andreola and S. E. Wheeler, *WIREs Computational Molecular Science*, 2021, **11**, e1510.
3. F. Neese, *WIREs Computational Molecular Science*, 2022, **n/a**, e1606.
4. C. Bannwarth, E. Caldeweyher, S. Ehlert, A. Hansen, P. Pracht, J. Seibert, S. Spicher and S. Grimme, *WIREs Computational Molecular Science*, 2021, **11**, e1493.

5. S. Grimme, F. Bohle, A. Hansen, P. Pracht, S. Spicher and M. Stahn, *The Journal of Physical Chemistry A*, 2021, **125**, 4039-4054.
6. N. Patil, A. Mavrandonakis, C. Jérôme, C. Detrembleur, J. Palma and R. Marcilla, *ACS Applied Energy Materials*, 2019, **2**, 3035-3041.
7. N. Patil, A. Mavrandonakis, C. Jérôme, C. Detrembleur, N. Casado, D. Mecerreyes, J. Palma and R. Marcilla, *J Mater Chem A*, 2021, **9**, 505-514.
8. L. Chen, J. L. Bao, X. Dong, D. G. Truhlar, Y. Wang, C. Wang and Y. Xia, *ACS Energy Letters*, 2017, **2**, 1115-1121.
9. J. Li, C. Han, X. Ou and Y. Tang, *Angewandte Chemie International Edition*, 2022, **61**, e202116668.
10. M. S. Chae, A. Nimkar, N. Shpigel, Y. Gofer and D. Aurbach, *ACS Energy Letters*, 2021, **6**, 2659-2665.
11. J. Bitenc, A. Scafuri, K. Pirnat, M. Lozinšek, I. Jerman, J. Grdadolnik, B. Fraisse, R. Berthelot, L. Stievano and R. Dominko, *Batteries & Supercaps*, 2021, **4**, 214-220.
12. T. Tojo, Y. Sugiura, R. Inada and Y. Sakurai, *Electrochim Acta*, 2016, **207**, 22-27.
13. A. L. Lipson, S.-D. Han, S. Kim, B. Pan, N. Sa, C. Liao, T. T. Fister, A. K. Burrell, J. T. Vaughey and B. J. Ingram, *J Power Sources*, 2016, **325**, 646-652.
14. J. Wang, S. Tan, F. Xiong, R. Yu, P. Wu, L. Cui and Q. An, *Chem Commun*, 2020, **56**, 3805-3808.
15. C. Chen, F. Shi, S. Zhang, Y. Su and Z.-L. Xu, *Small*, 2022, **18**, 2107853.
